# Integrating traditional indigenous medicine and western biomedicine into health systems: a review of Nicaraguan health policies and miskitu health services

**DOI:** 10.1186/s12939-015-0260-1

**Published:** 2015-12-14

**Authors:** Heather Carrie, Tim K. Mackey, Sloane N. Laird

**Affiliations:** Center for Health Policy and Leadership, Bastyr University , 14500 Juanita Drive NE, Kenmore, WA 98028 USA; University of California, San Diego – California Western School of Law, San Diego, USA; Department of Anesthesiology, University of California, San Diego School of Medicine, San Diego, USA; Division of Global Public Health, Department of Medicine, University of California, San Diego School of Medicine, San Diego, USA; Global Health Policy Institute, San Diego, California USAᅟ; University of California, San Diego – Extension, San Diego, USA

**Keywords:** Indigenous health, Traditional medicine, Health and human rights, Miskitu people, Global health governance, Nicaragua health policy

## Abstract

Throughout the world, indigenous peoples have advocated for the right to retain their cultural beliefs and traditional medicine practices. In 2007, the more than 370 million people representing 5000 distinct groups throughout the world received global recognition with the adoption of the United Nations Declaration on the Rights of Indigenous Peoples (UNDRIP). UNDRIP Article 24 affirms the rights of indigenous peoples to their traditional medicines and health practices, and to all social and health services. Although not a legally binding agreement, UNDRIP encourages nation states to comply and implement measures to support and uphold its provisions. Within the context of indigenous health and human rights, Nicaragua serves as a unique case study for examining implementation of UNDRIP Article 24 provisions due to the changes in the Nicaraguan Constitution that strive for the overarching goal of affirming an equal right to health for all Nicaraguans and supporting the integration of traditional medicine and biomedicine at a national and regional level. To explore this subject further, we conducted a review of the policy impact of UNDRIP on health services accessible to the Miskitu indigenous peoples of the North Atlantic Autonomous Region (RAAN). We found that although measures to create therapeutic cooperation are woven into Nicaraguan health plans at the national and regional level, in practice, the delivery of integrated health services has been implemented with varying results. Our review suggests that the method of policy implementation and efforts to foster intercultural collaborative approaches involving respectful community engagement are important factors when attempting to assess the effectiveness of UNDRIP implementation into national health policy and promoting traditional medicine access. In response, more study and close monitoring of legislation that acts to implement or align with UNDRIP Article 24 is necessary to ensure adequate promotion and access to traditional medicines and health services for indigenous populations in Nicaragua and beyond.

## Introduction

There are more than 370 million people representing 5000 distinct indigenous peoples throughout the world [1]. Within this diverse global community, a strong and growing movement continues to advocate for self-determination with the right to regain and retain traditional indigenous cultural practices. A steady rise of united influence emerged in the late 1970s and continues to gain momentum with the passing of international declarations and national laws [1]. Included in some of these international, national, and regional policies, are provisions calling for ensuring and protecting the rights of indigenous peoples to their traditional medicine. These policies are written with the intention of supporting both the traditional indigenous medicine healers in offering their services and for the indigenous individuals in accessing traditional medicines and practices [2].

In 2007, in a landmark moment following more than two decades of negotiation, 143 member states adopted the United Nations Declaration on the Rights of Indigenous Peoples (UNDRIP) reflecting widespread recognition by the international community to the individual and collective rights of indigenous peoples. UNDRIP Article 24 specifically affirms the fundamental rights of indigenous peoples to their traditional medicines and health practices, and to all social and health services. Although not a legally binding instrument, UNDRIP represents an important step forward in developing a framework of internationally agreed upon norms and principles specifically reaffirming the unique human right to health for indigenous peoples [2]. Though the UNDRIP was designed to promote a set of international standards and obligations to be adopted by member states and potentially translated into national policy making, there is a general lack of research examining its effectiveness and influence in the context of health policy.

Hence, this review seeks to determine if the principles enshrined in UNDRIP have been successfully translated and implemented into national health policy in Nicaragua, a country home to a Native American indigenous population known as the Miskitu peoples of the North Atlantic Autonomous Region (RAAN) [3]. In the latest population census conducted by the Nicaragua National Institute of  Information Development (INIDE) in 2005, Miskitus were found mainly living in the northeast corner of Nicaragua in a province known as the RAAN, with a population of more than 120,000 – nearly 45,000 urban and 75,000 rural [4]. The Miskitu peoples of Nicaragua were chosen as a focal point for this review because they are affected by both national and regional policies that include provisions for supporting the rights of indigenous peoples to their traditional medicine. The Miskitu possess a unique history in gaining semi-autonomy from the central government of Nicaragua after indigenous insurgency efforts against the Sandinista government led to the Autonomy Statute of 1987 (Law 28) initiated in hopes of bringing peace to the region. Although Law 28 recognizes the rights to self-determination for the Atlantic Coast people (mostly Miskitu), the Nicaraguan central government maintains influence in regional policy implementation - including those policies affecting health service accessibility [5].

## Review

In this review we specifically set out to assess whether Nicaragua’s national and regional health plans positively influence the integration of traditional indigenous medicine and western Biomedicine; how these policies impact the health services being accessed, delivered, and used by the Miskitu; and whether these policies could serve as a global health policy model for other indigenous populations who wish to promote their right to health as recognized by UNDRIP. To accomplish this, we conducted a literature review and analysis of documents related to Nicaraguan indigenous health, Nicaragua’s national health policy and laws, and health provisions of the UNDRIP. The inclusion criteria for documents reviewed in this study comprised of: peer-reviewed literature; reports published by NGOs and other civil society actors; and reports and policy documents issued by local, national, regional or international organizations discussing Nicaraguan indigenous health policy. In retrieving these documents we used a combination of online Google searches for keywords associated with Nicaraguan indigenous health, searched for literature on the subject from PubMed/Medline and Academic Search Complete databases, and also searched for documents in both English and Spanish on websites of international and regional organizations as well as from Nicaraguan government agencies. Document extraction and review was conducted from October to December, 2014.

Our review begins with a brief summary of global health governance for indigenous peoples' right to health and Nicaragua’s own national and regional health plans to discover how traditional knowledge and indigenous peoples practices are included as policy to be implemented in health care settings. We then examine the health services accessible to the Miskitu peoples and seek to understand how traditional practices and biomedicines are used separately or together to provide care for specific conditions. In addition, we examine what training exists for health care providers in this community to support inter-cultural respect for both traditional knowledge and medical empirical evidence. Lastly, we assess whether Nicaragua’s national and regional health plans meet the standards set forth under UNDRIP Article 24 and provide recommendations for future policy implementation.

### Brief history of global health governance for indigenous peoples’ right to health

It is important to examine other international policy instruments that have been adopted and that have provided critical foundational support for the right to health for Indigenous populations that preceded the adoption of the UNDRIP. In 1989, The International Labour Organization Convention, (No. 169) was the first contemporary international policy guaranteeing rights to indigenous peoples and specifically declares a right to health services for indigenous and tribal peoples in independent countries. In addition, the Convention calls for government responsibility in providing community-based, culturally appropriate care, preferably from health care providers employed from the local community, with input from the community [6].

In 1993 and 1997 , the Pan American Health Organization (PAHO) passed resolutions to promote the right to health and the access to health care for indigenous peoples in the Americas [7]. Resolutions CD37.R5 and CD40.R6: Health of Indigenous Peoples Initiative acknowledges the inequities in the status of health and lack of access to basic health services [7]. These resolutions consist of provisions to support the right to self-determination and respect for cultural values regarding health care along with the right to alternative models of care to deal with “insufficient coverage, inadequate access, and the lack of acceptability of health services on the part of indigenous populations” [7]. It also urges member governments to establish mechanisms that allow for representation of indigenous peoples in the development of health care services for their own populations [7]. The PAHO resolutions served to guide the health measures framework for indigenous peoples residing in the Americas [7].

In 2007, after a decade of additional PAHO resolutions and increased social and political activism to have indigenous peoples' rights acknowledged internationally, the United Nations drafted and adopted a Declaration on the Rights of Indigenous Peoples (UNDRIP) (143 states affirming, 4 voting against [Australia, Canada, New Zealand and the United States], and 11 abstentions). In this declaration, the right to health is clearly stated in Article 24, Section One by affirming the right to traditional medicine within indigenous health care practices and access to other health care services without discrimination and in Section Two by affirming the right to the highest level of mental and physical health with governmental support in obtaining this goal [2]. These rights are in accordance with the World Health Organization (WHO) Constitution’s declaration of health as a fundamental human right and the United Nations Economic and Social Council (ECOSOC) function to promote respect for human rights [8, 9]. 

### Nicaragua’s national health laws and indigenous health policy

Nicaragua’s national and regional laws closely parallel the trajectory of the body of international declarations and principles concerning indigenous peoples. However, the first Nicaraguan national law with provisions for indigenous peoples' rights was initiated two decades before UNDRIP with the adoption of the Nicaragua Constitution of 1987. Even preceding the 1987 Constitution, Nicaragua’s Ministry of Health created a program in 1985 to “revitalize popular and traditional medicine” [10] as a necessary response to the wartime high costs of imported pharmaceutical materials [10]. The Nicaragua Constitution Article 5 expresses that “the State recognizes the existence of indigenous peoples who enjoy the rights, … and guarantees … their identity and culture, to have their own forms of social organization and administer their local affairs, as well as to preserve the communal forms of land property…” [11]. Article 180 reinstates this idea by giving the communities of the Atlantic Coast, where most of the Miskitu reside, the right to live and create organizations based upon their cultural traditions [11].

Additionally, Article 89 of the Nicaragua Constitution supports the communities of the Atlantic Coast in the right to maintain and cultivate their own cultural identities, as well as to manage their local affairs within their traditions and historical customs. This article also demands that the state recognize a communal form of land-ownership within the Atlantic Coast. Thus, the Mistiku peoples living in the Atlantic Coast region have a form of land ownership to enable them to maintain and support conservation of traditional plants, animals and mineral medicines [11]. Hence, the right to manage and access their traditional medicines is also affirmed in Article 24 of the UNDRIP along with the right to self-determination at the state level [2].

To further revitalize access and promotion of traditional medicine, the Ministry of Health created the National Centre of Popular and Traditional Medicine in 1989 with the objectives of: organizing traditional medicine research; training health care providers in traditional medicine practices; and commercializing the production of medicinal plants. In 1991, the Centre separated from the Ministry of Health to become its own non-profit foundation with new objectives to recover and preserve traditional practices; promote the use of medicinal plants, and create a medicinal plant distribution network through pharmacies in the health system [10].

Although health care services for the indigenous peoples of Nicaragua initially were managed at the national level, this changed when the federal government developed the National Health Plan of 2004–2015, which started to decentralize medical services and resources from the national level to regional authorities. This decentralization of health care was influenced by international organizations calling for a sector-wide health care services delivery development approach. The plan also included provisions to allow for non-government organizations (NGOs) and health care foundations to set up programs within the country, as well as for the privatization of some health care services. Additionally, an aim of the policy was to incorporate or integrate the “cosmovision” of the communities into practice, to define the cultural-specific meaning of full wellness and complete health. The cosmovision approach originally began as a concept started in 1996 as part of Nicaragua’s health plan for the RAAN region to foster therapeutic cooperation in equal terms between traditional medicine practices and western biomedicine [3].

To further support the cosmovision of indigenous communities, Nicaragua’s General Health Law of 2005 calls for indigenous peoples within the Atlantic Coast regions to be enabled to develop health methods that are consistent with their traditions and communities. Specifically, provisions state that the RAAN region may define and implement through regulations models of health care according to their traditions and customs of medicine and designate health authorities by methods they adopt [5]. This law is consistent with the PAHO initiatives, which demands the respect for indigenous peoples values and social organizations [7].

With the 2007 national election, a change in government guided health policy into a different direction. The decentralization remained, but the new health plan moved away from privatization and toward a policy based on the concept of universal care with ideas of free access to basic services. This new direction in health policy was based on concepts of equity and solidarity, with the idea of citizen responsibility and participation in their health care [12]. The policy was deemed to be community-based, which is a provision of the International Labour Organization (ILO) Convention 169, for indigenous and tribal peoples, and was ratified by Nicaragua in 2010 [6]. The plan also placed an emphasis on families. In 2009, the Modelo de Salud Familiar y Comunitario (MOSAFC), (Model of Family and Community Health) supported the idea that health care should incorporate the concept of an integrated approach with a focus on the promotion of health that addresses the differences within health determinants by including traditional medicine with western biomedicine [12]. This concept of integrating health care is nation-based, as it does not consider global policies, but tries to address local issues of the Atlantic Coast region and the multiple communities that have developed overtime with an emphasis on their unique customs and cultures.

In 2011, Nicaragua enacted *Ley No. 759, Ley de Medicina Tradicional Ancestral* (Law No. 759, Law of Traditional Ancestral Medicine), which specifically addresses access and equity to traditional and ancestral medicine [13]. The law also reestablished coordination between regions of indigenous peoples, updated a project plan for construction of an alternative medicine center, and has led to coordination between the Ministry of Health to incorporate intercultural concepts into health care models [13].

Recently, in 2014, Nicaragua adopted Nicaragua adopted *Ley No. 774, Ley de Medicina Natural, Terapias, Complementarias Y Productos Naturales En Nicargaua *(Law No.774, Law of Natural and Complementary Medicine, Therapies, and Natural Products), which establishes provisions to promote access and use of natural medicine that includes traditional medicine practices, products and health services. Additionally, Law 774 declares the right of choice in therapeutic care to the Nicaraguan people and outlines regulatory procedures to oversee health care providers, products, and services [14]. Articles 23 and 24 specifically address safety in the use of medicinal plants with a cautionary protocol for toxic botanicals and guidelines for regulation of use. Finally, the law supports both the integration of natural medicine into the national health system and appointment of representatives of natural medicine organizations into the National Health Council [14].

### Gaps in translation and implementation of international law and domestic Nicaraguan indigenous health policies

In reviewing Nicaragua’s national and regional laws pertaining to health and health care services, the provisions appear to generally align with the standards set forth in UNDRIP, but nevertheless introduce significant policy gaps and challenges that require further examination. Specifically, actual health care accessibility and health services delivery for indigenous communities in Nicaragua appears to be deficient, indicating that translation of UNDRIP into national health policy exists, but actual implementation to ensure indigenous health care rights and use of traditional medicine has not yet been adequately effectuated in these communities.

Under the Nicaraguan health system, accessing basic health services, a provision under UNDRIP Article 24 and included in the national health plan, remains difficult for Mistiku communities due to lack of public infrastructure (i.e. roads and affordable public transportation in the region) as well as a lack of sufficient health care workers to cover services needed to ensure access to hospitals and other treatment facilities [3]. Most health care services targeted towards these populations are delivered by health posts that consist of limited health care staff coverage (e.g. only one doctor and two nurses) [3]. Another method to deliver health care services to the Mistiku, which was developed under the community-based plan, allows for medical brigadistas (also known as independent health volunteers) to serve the rural areas of the Atlantic Coast, in order to promote public health interventions. Yet, these health care providers may not have the cultural knowledge or experience to appropriately deliver health care services or critical health behavior-related information, including inability to speak or understand the Mistiku language. Despite these challenges, in the context of basic access to health care, this indigenous community is able to access medical services at the two hospitals located in the autonomous regions or in other places of the country, though the delivery of such services may not integrate indigenous medicine [15].

Another provision of the UNDRIP Article 24, Section One, calls for the conservation of plants, animals, and minerals used in traditional medicine [2]. As previously described, the Nicaraguan Constitution includes provisions for these protections in the form of communal land-ownership rights, which was included in the country’s Constitution as a means to enable indigenous peoples to protect their land, property and resources [11]. However, the Constitution nor the national laws that implement these principles, do not specifically create an adequate mechanism (whether a legal process, dispute resolution system, or other form of due process) to sufficiently protect these resources from outside sources or the government itself, essentially leaving the Miskitu and others without the appropriate means to exercise their rights and protect their resources either for the use of traditional medicine or for conservation purposes.

### Integration effort: traditional medicine and western biomedicine

Although the intention to create therapeutic cooperation between indigenous traditional healers and western biomedical health care providers is woven into Nicaraguan health plans at the national and regional level, in practice integrated health services delivery has been implemented for the RAAN Miskitu with varying efforts and results [3, 16]. The more successful integration efforts foster intercultural cooperation and a “from the community and for the community” [16] approach whereas the less successful integration efforts treat traditional medicine as mostly a supplementary or last resort resource. 

In a 2009 study, Wedel reviewed existing literature that examined the collaborative efforts of traditional healers and biomedical health care providers serving the RAAN Miskitu population, noting that the biomedical providers often had an advantage over traditional healers in their authority and use of treatments [3]. He found traditional medicine care was primarily used at the discretion of biomedical health care providers as part of their own therapeutic treatment or alternatively by a traditional healer called in only to care for those who suffered with ailments outside of the general scope of biomedicine [3]. With illnesses deemed “spiritual” or “cultural,” Wedel found a willing transfer of medical authority from the biomedical providers to the traditional healers for therapeutic treatment; that Miskitu healers were used as a marginalized complement mostly when biomedicine failed [3].

This last-resort use of traditional medicine undermines the essence of a collaborative approach in integrating health services for therapeutic cooperation, and instead positions the western biomedical health provider as the medical authority acting as a gatekeeper to the traditional healers. When interviewed by Wedel, many Miskitu traditional healers expressed an openness to working with the western biomedical community to share in the care and clinical management of patients. Some also expressed a frustration with those western physicians who completely dismiss the value of traditional medicine and refuse to consult with the local healers, especially when the illness shows signs of being rooted in sorcery or of a spiritual nature [3]. When interviewing biomedical providers, Wedel found that those who were born in the RAAN region or were of Miskitu origin were more open to traditional medicine, and that western nurses were more accepting of the traditional practices than western doctors [3].

### Barriers to implementing integration efforts

The most evident barrier to implementing integration efforts is the vastly different belief systems of western biomedical practitioners and their traditional indigenous healer counterparts in regards to illness, health and healing. Much of the Miskitu health beliefs involve a connection between spirit entities and illness [3]. Hence, those western biomedical practitioners unfamiliar with Miskitu culture or skeptical of these beliefs, may discount the value of traditional healers. Likewise, traditional healers who may have attained most of their medicinal knowledge through apprenticeship or non-university training, may not comprehend or believe in the western health care model. These divergent educational backgrounds and belief systems can be further complicated by potential language barriers inhibiting the success of working together and collaboratively on behalf of shared patients [3].

Additionally, funding for traditional medicines can be limited and can hamper integration efforts. Specifically, regional Health Ministries often claim that there is significant difficulty in planning and budgeting for some health services provided by traditional healers who offer an individualized approach to treatment that can require varying remedies and fees in contrast to standardized health services offered by western biomedical personnel covered by the Health Ministry as basic health services [3]. With funds being distributed so unevenly between the health providers, biomedical practitioners may have more access to health systems financing in addition to their general advantage of authority in clinical decision-making [3]. In this sense, though the overall cost of delivery for traditional medicines may be lower than the price of modern medicine, lack of sufficient funding and coverage to enable access may limit the use of traditional medicine in communities where it is desired. Further, there is also the need to better integrate traditional medicine and modern medicine in order to address broader public health and patient safety concerns that may arise from their uncoordinated use - such as the problems of abuse/overdose, toxicity or poison risk, potential for adverse events, medication interaction, overlap of active ingredient and need for appropriate dosaging - that have not been fully explored for potential benefit.

### Efforts to foster therapeutic cooperation

In spite of these barriers, efforts to foster therapeutic cooperation and collaboration between health care providers continues through inter-cultural training, integrated participation in therapeutic encounters, third party links and increasing the number of Miskitu health care providers with an education in biomedicine. As an example, the local university URACCAN (Universidad de las Regiones Autónomas de la Costa Caribe de Nicaragua) hosts the Institute of Traditional Medicine and Community Development (IMTRADEC) serving as a link between the national health policy plan, traditional healers and the Health Ministry by organizing educational workshops and multidisciplinary health brigades [3]. These workshops focus on inter-cultural exchange with courses on traditional medicine and discussions on health policies. Those working for IMTRADEC also facilitate opportunities for biomedical practitioners and traditional healers to work directly together in the provisioning of health care and sharing knowledge for the benefit of their shared patients.

Working more closely with local communities, Acción Médica Cristiana (AMC), a Nicaraguan ecumenical non-governmental health and development agency is attempting to effectively address social determinants of health directly with community members through a method of Participatory Action Research (PAR). This process requires community members to prioritize their own needs for addressing the determinant of health problems such as disease outbreak, food security, safety, and health impacts of natural disasters in order to effectively create a community health system based on the cultural values of the community [16]. In this collaborative approach, AMC workers learn more about traditional medicine practices and the cultural views of the community to successfully work in therapeutic cooperation with empowered community members [16]. Hence, efforts by the AMC represent an example of successful community engagement that results in a community based approach to addressing the social determinants of health and a more collaborative integration of traditional medicine and biomedicine health services delivery.

At the international level, the WHO Traditional Medicine Strategy 2014–2023 outlines strategic objectives for Member States to further the integration of traditional medicine into national health systems and to protect consumers of care through the regulation of products, practices and practitioners. Strategic Objective 4.2.1 specifically addresses the need for creating a regulatory model to ensure the safety and effectiveness of products and services. With a goal to support diverse Member States in creating unique regulatory frameworks to “meet the health needs and choices of their people”, [17] WHO encourages collaborative partnerships between regional and local stakeholders in addition to setting international standards to guide national policy [17].

## Conclusion

In summary, our review indicates that although Nicaragua has embarked on a health policy strategy that includes core principles of the UNDRIP with the intention to recognize the fundamental right to health for indigenous communities such as the Miskitu, full implementation of the UNDRIP and the ability to ensure effective therapeutic cooperation between indigenous traditional healers and western biomedical health care providers remains challenging for a variety of different reasons. As of 2009, IMTRADEC appeared to be the only organization devoted to the implementation of the national and regional plans to support the Miskitu indigenous peoples in securing their right to their traditional medicines and fostering the integration of health services. This may have been influenced by a continuing dominance of western biomedicine over traditional medicine in Miskitu health care practice settings and health systems financing. Lack of policy implementation and prioritization is occurring despite the fact that Nicaragua had taken significant steps to translate the general principles of UNDRIP into national and regional health policy, though more progressive constitutional amendments supporting equality of all health care providers would likely be helpful. In response, more robust efforts to fully support integration of health care practices and establishment of an adequately funded inter-cultural training program for all health care providers and healers must continually be implemented to create a blended health care service system and cross cultural therapeutic cooperation. Further, international development assistance within the health sector needs to recognize the importance of supporting therapeutic cooperation and community cosmovision as an integral part of implementing international indigenous health rights that can also lead to better health care access and outcomes. In this sense, the future of Nicaragua’s national indigenous health policies is critical to the current discourse of indigenous global health governance, policy, and international human rights law requiring further study, advocacy and support in order to ensure the realization of internationally agreed upon principles respecting traditional medicine and the right of indigenous peoples to the highest level of mental and physical health.

## Data availability

We reviewed publicly available data that is available per the references included in this study.

## Abbreviations

AMC: Acción Médica Cristiana
ECOSOC: United Nations Economic and Social Council
ILO: International Labour Organization
IMTRADEC: Institute of Traditional Medicine and Community Development
MOSAFC: Community and Family Health Model
NGO: Non-Government Organizations
PAHO: Pan American Health Organization
PAR: Participatory Action Research
RAAN: North Atlantic Autonomous Region
UNDRIP: United Nations Declaration on the Rights of Indigenous Peoples
URACCAN: Universidad de las Regiones Autónomas de la Costa Caribe de Nicaragua
WHO: World Health Organization
