# Erratum to: Integrating traditional indigenous medicine and western biomedicine into health systems: a review of Nicaraguan health policies and miskitu health services

**DOI:** 10.1186/s12939-015-0293-5

**Published:** 2016-02-03

**Authors:** Heather Carrie, Tim K. Mackey, Sloane N. Laird

**Affiliations:** Center for Health Policy and Leadership, Bastyr University, 14500 Juanita Drive NE, Kenmore, 98028 WA USA; San Diego – California Western School of Law, University of California, San Diego, USA; Department of Anesthesiology, San Diego School of Medicine, University of California, San Diego, USA; Division of Global Public Health, Department of Medicine, San Diego School of Medicine, University of California, San Diego, USA; Global Health Policy Institute, San Diego, California USA; University of California, San Diego – Extension, San Diego, USA

Unfortunately, the original version of this article [1] contained several mistakes. In the first affiliation, an errant space was introduced after “Bastyr University”. The line reading “access and equity to traditional and ancestral medicine” should instead have read “equity and access to traditional and ancestral medicine”. In the line reading, “Recently, in 2014, Nicaragua adopted Nicaragua adopted”, the term “Nicaragua adopted” was mistakenly repeated. The line that reads “autonomous regions or in other places of the country” should read “autonomous regions or other regions of the country”. The acknowledgements section was also missing the following acknowledgement, “This research was supported by the Joint Masters Program in Health Policy and Law Capstone Student Scholarship and authors greatly acknowledge this support”. The original article has also been updated to correct these errors.
